# Robot‐assisted laparoscopic continent cutaneous urinary diversion in adults: A single‐centre study

**DOI:** 10.1002/bco2.449

**Published:** 2024-10-30

**Authors:** Thomas Loubersac, Etienne Lavallée, Benédicte Reiss, Marc Lefort, Pierre Kieny, Marc‐David Leclair, Jérome Rigaud, Loic Le Normand, Brigitte Perrouin‐Verbe, Chloé Lefevre, Marie‐Aimée Perrouin‐Verbe

**Affiliations:** ^1^ Department of Urology and Pediatric Urology Nantes Université, Centre Hospitalo‐Universitaire de Nantes Nantes France; ^2^ Department of Pediatric Urology Nantes Université, Centre Hospitalo‐Universitaire de Nantes Nantes France; ^3^ Oncology Division, Department of Surgery, Faculty of Medicine Centre Hospitalier Universitaire (CHU) de Québec Research Centre, Université Laval QC Québec Canada; ^4^ Physical Medicine and Rehabilitation Department Nantes Université, Centre Hospitalo‐Universitaire de Nantes Nantes France; ^5^ Department of Urology Nantes Université, Centre Hospitalo‐Universitaire de Nantes Nantes France; ^6^ INSERM U 1235, The Enteric Nervous System in Gut and Brain Disorders Nantes Université Nantes France

**Keywords:** continent urinary diversion, laparoscopy, Mitrofanoff procedure, neurogenic bladder, robotic surgery

## Abstract

**Objectives:**

To show that robot‐assisted laparoscopic cutaneous continent urinary diversion (RALCCUD) is feasible and safe; however, data on clinical outcomes in adults are lacking.

**Materials and methods:**

We conducted a retrospective study of all adults who underwent RALCCUD between 2017 and 2022 at a single tertiary reference centre.

Patient characteristics, clinical information and perioperative outcomes were recorded. All patients underwent pre‐ and postoperative urodynamic evaluations.

Functional outcomes were evaluated at 3 months, then yearly. Continence was defined as no stomal or urethral leakage.

**Results:**

Twelve patients, mostly women (*n* = 11), median (IQR) age 47.4 (19–57) years underwent RALCCUD (four Mitrofanoff, four Yang‐Monti and four Casale). The main indication for surgery was inability to perform intermittent self‐catheterization through the native urethra.

Eleven patients (92%) had neurogenic lower urinary tract disease caused by spinal cord injury or spinal dysraphism.

Median (IQR) operative time was 313 (285–367) min. Four patients (33%) underwent concomitant procedures: three supratrigonal cystectomy (SC) with augmentation cystoplasty (AC) and one artificial urinary sphincter (AUS). No conversions to an open approach were required. Median (IQR) follow‐up was 51 (40–61) months. One early postoperative complication occurred (Clavien grade III). The late postoperative complication rate was 17%, with three complications occurring in three patients.

At the last follow‐up, all patients could self‐catheterize through the tube, and the stomal and urethral continence rate was 100%.

**Conclusion:**

RALCCUD is feasible and safe in adults, with a high rate of stomal and urethral continence and a low complication rate.

**Abbreviations and Acronyms**
AC: augmentation cystoplasty
AUS: artificial urinary sphincter
BMI: Body Mass Index
BPS/IC: bladder pain syndrome/interstitial cystitis
CCUD: continent cutaneous urinary diversion
ISC: intermittent self‐catheterization
NDO: neurogenic detrusor overactivity
NLUTD: neurogenic low urinary tract dysfunction
RALCCUD: robot‐assisted laparoscopic continent cutaneous urinary diversion
SC: supratrigonal cystectomy
SCI: spinal cord injury

## 1 INTRODUCTION

Guidelines for the treatment of people with neurogenic low urinary tract dysfunction (NLUTD) include the management of neurogenic detrusor overactivity (NDO) combined with intermittent self‐catheterization (ISC)^1^ when they are unable to empty their bladder. This treatment strategy aims to protect the upper urinary tract and maintain urinary continence by ensuring a low‐pressure bladder reservoir.^1^, ^2^, ^3^ However, some people who require ISC are unable to perform it through the native urethra because of upper‐limb disability, difficulty reaching or finding the urethra or urethral destruction. Cutaneous continent urinary diversion (CCUD) (Mitrofanoff,^4^ Yang‐Monti^5^ or Casale^6^ procedures) can be offered to such individuals to allow ISC. Several case‐series studies have reported good long‐term results from this technique in children^7^ and adults.^8^

Robot‐assisted laparoscopic cutaneous continent urinary diversion (RALCCUD) was developed in the 2000s^9^ and provides the advantage of shorter length of hospital stay compared to the open approach.^10^ Several studies with long‐term follow‐ups have demonstrated the feasibility and efficacy of (RALCCUD) in children,^11^, ^12^ but only a few small case‐series have been published in adults.^13^, ^14^

Despite these advances, there is still a need to evaluate the postoperative complications and functional outcomes of this robotic technique in adult centres.

Our aim is to evaluate the efficacy of RALCCUD and to report perioperative outcomes and preliminary functional outcomes in terms of continence, catheterization and reoperation during the follow‐up period.

## 2 METHODS

We conducted a single centre, retrospective, case‐series study of individuals who underwent RALCCUD in our department between 2017 and 2022. Ethical approval was granted by our local ethical committee the Institutional Ethical and Clinical Research at Nantes Université (Groupe Nantais d'Ethique dans le Domaine de la Santé) review board (Nantes, 2022) number ‘AVIS 22‐3‐170’.

Indications for the surgery were the inability to perform ISC through the urethra because of difficulty reaching or finding the urethra. Inclusion criteria for the study were age at least 15 years old and with a postsurgical follow‐up of at least 3 months.

Before surgery, all individuals underwent an assessment by a multidisciplinary team that included at least a urologist, a physical medicine and rehabilitation doctor and, if necessary, an occupational therapist. The ability to hold a catheter and to self‐catheterize through an abdominal stoma was assessed during a short hospital stay prior to surgery.

Individuals with traumatic spinal cord injury underwent MRI to rule out the presence of syringomyelia, which may be a contra‐indication to the laparoscopic approach.^15^

An assessment was performed preoperatively, at 3 months postoperatively and then yearly. The assessment included stomal and urethral continence, satisfaction, renal function and urodynamic function, outcomes and complications.

The primary outcome was continence status at the last follow‐up. Continence was defined as no leakage (no pad) from either the urethra or the stoma without the need for secondary incontinence surgery after RALCCUD.^16^ The secondary outcomes were the rate of clinically significant postoperative complications (≥grade 3 on the Clavien–Dindo classification^17^), classed as early (0–30 days postoperative) or late (>30 days postoperative), and the rate of stomal complications requiring reintervention. The stomal complications requiring reintervention included stenosis, false route and incontinence after excluding other causes such as bladder overactivity.

### 2.1 Statistical analysis

Continuous variables are expressed as medians and interquartile ranges (IQRs; 25th and 75th percentiles) and categorical variables as numbers and percentages. Mann–Whitney test or Student's *t*‐test was used to compare continuous variables according to normal distribution (Shapiro–Wilk's test) and chi‐square or Fischer's exact test for categorical variables. A *p* < 0.05 was considered significant. SAS software (version 9.4, NC, USA) was used.

### 2.2 Surgical technique

All the procedures were performed by two surgeons.

The patient was placed in a modified lithotomy position with 25° of Trendelenburg to shift the bowel away from the pelvis. An 18Fr Foley catheter was placed in the bladder.

The camera port (8 mm) was placed intraperitoneally via an open laparoscopy approach under the umbilicus, and pneumoperitoneum was established with 12 mmHg insufflation pressure. An 8‐mm robotic port was placed on each side of the camera port, and a fourth robotic port was placed in the right iliac fossa at the same level. The ports were placed at least 8 cm apart. An additional 12‐mm port was inserted in the left iliac fossa for the assistant. After trocar placement, the robot (four‐arm Da Vinci Xi Surgical System®, Intuitive Surgical, Inc., Sunnyvale, CA, USA) was docked.

Each step of the procedure is described in the video (*Supplementary Material*). We have described the creation of the Mitrofanoff conduit for patient case 12.

### 2.3 Bladder mobilization

The bladder was freed from the peritoneum and mobilized by sectioning the urachus and the two umbilical arteries. It is critical to ensure that the bladder is adequately mobilized and that it can easily be brought to the anterior abdominal wall near the camera port.

### 2.4 Catheterizable tube preparation

The preferred choice for tube formation was the appendix. It was identified after mobilizing the caecum, from which it was then separated with care taken to preserve the blood supply.

Then, the length of the tube and the anatomical possibility to reach the planned stoma location were verified. A key issue for RALCCUD is an accurate evaluation of the distance between the implantation of the conduit in the bladder and the stomal anastomosis.

The tip of the appendix was sectioned, and a 14 Fr feeding‐tube was introduced to verify the patency of the whole appendix.

In case of previous appendectomy, or an unusable appendix, it is possible to use a retubularized tube, according to the Yang‐Monti technique.^5^ For this, a 3/0 polyglotone suture was placed on the ileum 30 cm from the ileocecal valve. The robot was undocked and performed a 4 cm incision below the umbilical incision. A 2 cm intestinal segment located 30 cm from the ileocecal valve was isolated extracorporeally to construct a retubularized tube.

Then the tube was reintroduced in the abdomen, and the robot was docked to carry on the surgery intraperitoneally. If the distance between the bladder and abdominal wall was long, a tube was prepared according to the Casale Principle,^6^ after isolation of an ileal segment of 3.5 cm in length.

In case of concomitant augmentation cystoplasty (AC), a 30‐cm ileal segment was isolated at 30 cm from the ileocecal valve at the same time.

### 2.5 Anti‐reflux anastomosis

**FIGURE 1:**
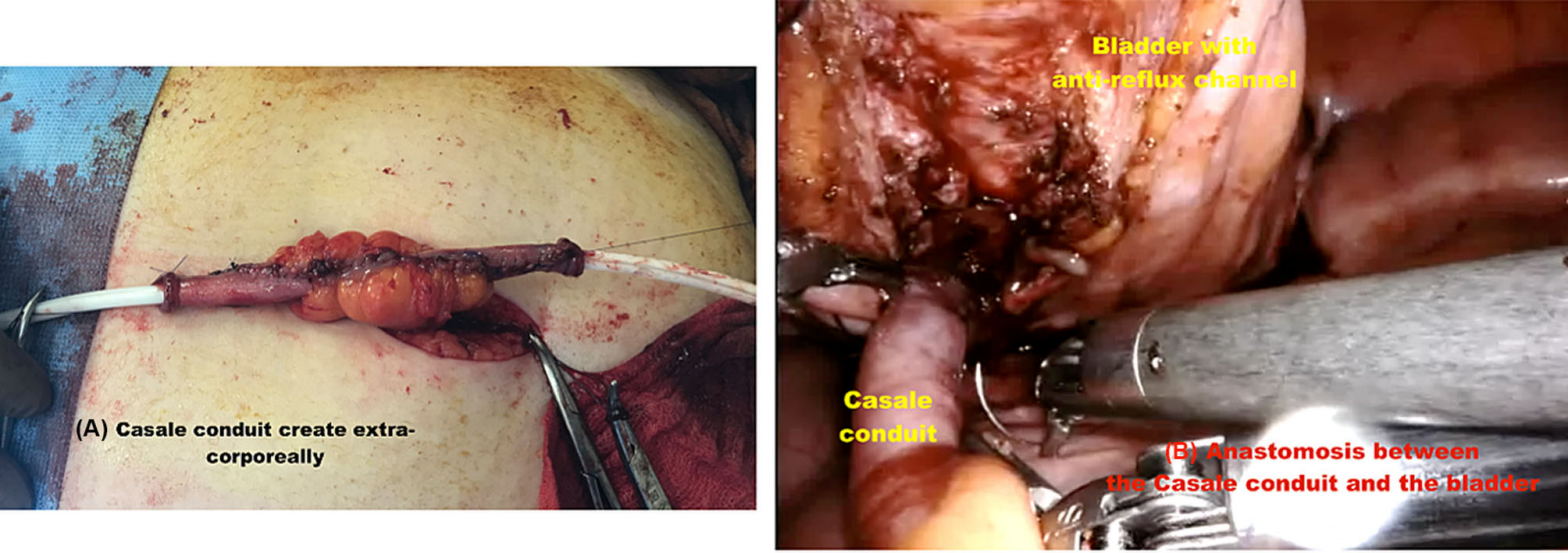
Creation of the anti‐reflux channel without augmentation cystoplasty: (A) Casale conduit create extra‐corporeally. (B) Anastomosis between the Casale conduit and the bladder.

The tube was always implanted in the posterior bladder wall. If concomitant AC was not performed, the tube was implanted according to the Lich‐Gregoir anti‐reflux principle. The bladder was filled with 300 mL of saline solution. The posterior wall of the bladder was opened sagittally with preservation of the bladder mucosa. Each side of the detrusor muscle was suspended to the anterior abdominal wall using a 3/0 polyglactin suture to provide adequate exposure (Figure 1). The catheterizable channel was sutured to the bladder mucosa with interrupted 5/0 polydioxanone sutures and the detrusor was closed with 3/0 polyglactin to create a submucosal anti‐reflux mechanism at least 4 cm long.

**FIGURE 2:**
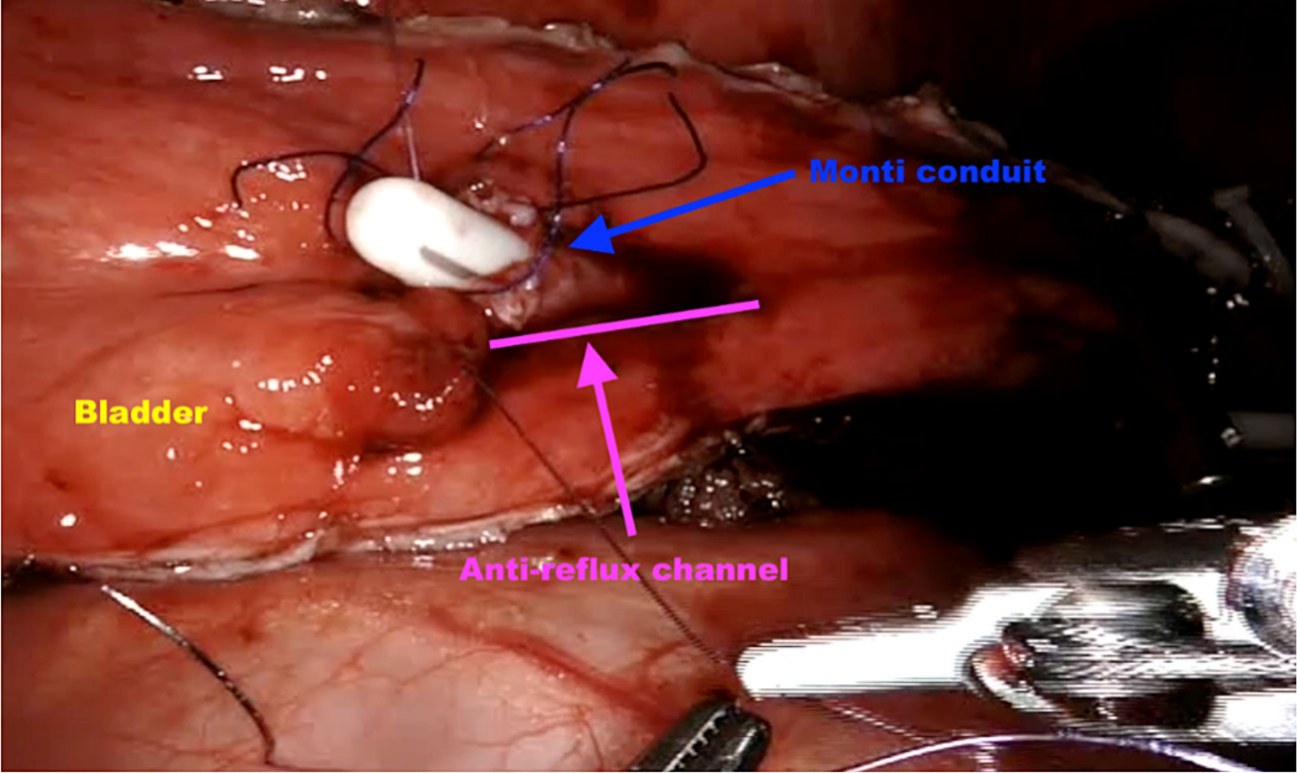
Creation of the anti‐reflux channel with augmentation cystoplasty: anastomosis between a Monti conduit and the bladder through a Leadbetter‐Politano anti‐reflux channel.

If concomitant AC was performed, a supratrigonal cystectomy (SC) was performed with preservation of the posterolateral bladder‐wall flap in which the efferent tube could be implanted according to the Politano‐Leadbetter principle (Figure 2), as previously published, using an open approach.^18^, ^19^

Then the AC was sutured to the remaining bladder using 3/0 V‐Lock running sutures.

The watertightness was checked by filling the bladder with 180 mL saline solution.

### 2.6 Stoma

The stoma was always placed at the umbilical level.

Before the anastomosis between the channel and the skin was performed, the bladder was sutured to the anterior abdominal wall with interrupted 2/0 sutures to avoid kinking of the channel and to minimize the extravesical portion as described for the open technique.^20^

The distal efferent tube was sutured to the skin with a V‐shaped skin flap using interrupted 4/0 polydioxanone sutures.

A 14Fr Foley catheter was then placed in the tube as well as in the urethra, for 21 days.

## 3 RESULTS

### 3.1 Patient characteristics and perioperative data

Patient characteristics and outcomes are shown in Table 1.

**TABLE 1:** Patient characteristics and perioperative data.

Case number	Age, years	Sex	Diagnosis	Type CUD	If no Mitrofanoff why (appendectomy or appendix too short or appendix obstruction)	Concomitant procedures	Operating time (min)	Blood loss (mL)	Stoma placement	Hospital stays (days)	Bowel activity recovery (in days)	Follow‐up, months	Immediate postoperative complications/Clavien grade
**1**	22	Female	NLUTD and BPS	Yang‐Monti conduit	appendix obstruction	SC and AC	392	200	Right iliac fossa	12	7	34	‐
**2**	17	Female	Spina bifida L2	Mitrofanoff APV	NA	SC and AC	628	150	Umbilicus	10	6	78	‐
**3**	51	Female	NLUTD due to SCI C5	Yang‐Monti modified Casale conduit	Appendix too short	SC and AC	643	200	Umbilicus	16	6	71	Wound dehiscence/1
**4**	67	Female	NLUTD due to SCI T10	Yang‐Monti conduit	Appendectomy	AUS	445	100	Umbilicus	11	5	63	Wound abscess/2
**5**	52	Female	NLUTD due to SCI T9	Yang‐Monti modified Casale conduit	Appendix obstruction	‐	285	100	Umbilicus	7	3	61	‐
**6**	17	Female	NLUTD due to SCI C6	Mitrofanoff APV	NA	‐	322	100	Umbilicus	7	4	5	Pre‐vesical abscess/2
**7**	20	Female	NLUTD due to SCI C3	Mitrofanoff APV	NA	‐	264	100	Umbilicus	14	7	55	Pre‐vesical abscess/2
**8**	73	Female	NLUTD due to SCI T4	Yang‐Monti modified Casale conduit	Appendectomy	‐	300	200	Umbilicus	8	3	55	Wound abscess/3
**9**	67	Female	NLUTD due to SCI C7	Yang‐Monti conduit	Appendectomy	‐	356	100	Umbilicus	7	3	46	‐
**10**	55	Male	NLUTD due to SCI T12	Yang‐Monti conduit	Appendectomy	‐	313	100	Umbilicus	8	3	45	‐
**11**	43	Female	NLUTD due to SCI C7	Yang‐Monti modified Casale conduit	Appendectomy	‐	367	100	Umbilicus	7	4	41	‐
**12**	18	Female	NLUTD due to SCI C5	Mitrofanoff APV	NA	‐	221	50	Umbilicus	11	5	25	‐
**Median Value** **(Min;Max)**	47.4 (17;73)						313 (224;643)	100 (50;200)		8 (7;16)	4 (3;7)	51 (5;78)	

Abbreviations: AC, augmentation cystoplasty; AUS, artificial urinary sphincter; BPS, bladder pain syndrome; CCUD, cutaneous continent urinary diversion; NLUTD, neurogenic low urinary tract dysfunction; NA, not applicable; SC, supratrigonal cystectomy; SCI, spinal cord injury.

Twelve patients were included; median (IQR) age was 47.4 (19.5–57.6) years (range 17–74 years), and 11 patients were female (92%). **The median (IQR) height was 167 (160–171) cm (range 143–195), the median (IQR) weight was 76 (60–93) kg (range 42–110), and the median (IQR) BMI was 26 (21–32) (range 17–41).**

Eleven patients had NLUTD caused by spinal cord injury (SCI) or spinal dysraphism, and one had a history of bladder pain syndrome/interstitial cystitis (BPS/IC). None had renal insufficiency before or after the surgery.

Four patients underwent a concomitant procedure: three SC with AC for poor bladder compliance, refractory NDO or refractory severe IC. One female with SCI was provided with an artificial urinary sphincter (AUS) for stress urinary incontinence related to neurogenic intrinsic sphincter deficiency.

Conversion to an open approach was not required for any patient. The median (IQR) operating time was 313 (285–367) min (range 224 to 643), and median (IQR) estimated blood loss was 100 mL (100–150).

The median (IQR) length of hospital stay for the 12 patients was eight^7^, ^8^, ^9^, ^10^, ^11^ days and was 13 days (range 10 to 16) for those who underwent concomitant procedures (*p* > 0.05).

The median operating time was 635 (range to 392–643) min for the patients who underwent concomitant procedures (SC and AC or AUS) and was 300 min (range to 224–367) for those who did not (*p* = 0.03).

### 3.2 Postoperative complications

The median (IQR) follow‐up duration was 51 (40–61) months (range 5 to 78).

The early and late complication rates were 8% (1/12) and 17% (2/12), respectively. The stomal complication rate was 17% (2/12) with a complication rate of 25% for Mitrofanoff, 25% for Casale conduit and 0% for Yang‐Monti. Details of the complications are provided in Table 2. No complications were rated >grade 3 on the Clavien–Dindo classification.

**TABLE 2:** Complications, treatments and outcomes.

Case number	Age (years)	Gender	Aetiology	Conduit	Operating time (min)	Blood loss (mL)	Stoma placement	Hospital stay (days)	Bowel activity recovery (in days)	Type of complication	Clavien grade	Apparition of complications (months)	Treatment	Outcome
**2**	17	Female	Spina bifida L2	Mitrofanoff APV	628	150	Umbilicus	10	6	Stomal stenosis and false route	3	35	Endoscopic dilatation	CIC via stoma, continent
**7**	20	Female	NLUTD due to SCI C3	Mitrofanoff APV	322	100	Umbilicus	7	4	Stomal incontinence	2	11	Switch from 200ui to 300ui injection of botulinum toxin type A (Botox®)	CIC via stoma, continent
**8**	73	Female	NLUTD due to SCI T4	Yang‐Monti modified Casale conduit	264	100	Umbilicus	14	7	Wound abscess	3	1	Surgical management	Healing of the wound
**8**	73	Female	NLUTD due to SCI T4	Yang‐Monti modified Casale conduit						Stomal incontinence	3	10	Polydimethylsiloxane endoscopic injection twice at the distal end of the conduit	CIC via stoma, continent
**8**	73	Female	NLUTD due to SCI T4	Yang‐Monti modified Casale conduit						Urethral incontinence	3	10	Peri‐urethral balloons pro‐ACT	CIC via stoma, continent
**Median (Min;Max)**	20 (17;73)				446 (264;628)	125 (100;150)		12 (7;14)	6 (4;7)			10 (1;35)		

Abbreviations: ACT, adjustable continence therapy; ISC, intermittent self‐catheterization; NLUTD, neurogenic low urinary tract dysfunction; SCI, spinal cord injury.

One patient (case 1) with a history of BPS/IC underwent a cystectomy with ileal conduit 34 months after RALCCUD because of refractory chronic pelvic pain. The patient was continent and had no catheterization difficulties before this reoperation.

Another patient (case 6) died from her initial disease (medullar glioblastoma) nearly 5 months after the surgery. No incontinence or catheterization difficulties were reported.

### 3.3 Functional results

The overall continence rate was 92% (11/12). The stomal continence rate was 92% (11/12). One patient (case 8) had stomal incontinence after insertion of a Casale conduit, which was treated by a bulking agent injection (Macroplastique® [Uroplasty] Laborie, Ontario, Canada). The urethral continence rate was 92% (11/12); the same patient (case 8) had urethral incontinence that was treated with a peri‐urethral balloon (Adjustable Continence Therapy, ACT®) (Table 2). At the last follow‐up, all patients reported stomal and urethral continence and performed ISC through the tube. No false passage or difficulty catheterizing the tube was reported.

The overall reoperation rate (including to treat complications and improve continence) was 33% (4/12) (Table 2). Only one stomal stenosis occurred at 35 months (case 2) and was managed by endoscopic dilatation.

No patient had renal insufficiency before or after the surgery.

The median (IQR) preoperative urodynamic characteristics were a bladder capacity of 475 (300–500) mL, a urethral closure pressure of 55 (37.5–120) cmH2O and bladder pressure at end of the filling of 21 (19–27) cmH2O. At the last follow‐up, the median (IQR) postoperative bladder capacity was 400 mL (350–500) with a bladder compliance >20. The bladder capacity of the three patients with an AC increased up to 300 mL and bladder compliance was >20 in all 3.

## 4 DISCUSSION

This study showed that all 12 patients who underwent RALCCUD were continent at the last follow‐up. Two patients experienced stomal complications and none experienced complications >Clavien 3.

These data complete those from a study in children that found RALCCUD to be safe and comparable in terms of long‐term functional outcomes with the results of traditional surgical procedures.^10^

The arrival and development of robotic surgery was a watershed moment in the world of laparoscopic surgery. It led to the expansion of the laparoscopic approach to complex neuro‐urology surgeries. Since the first RALCCUD reported in 2004,^9^ the technique has been developed and standardized in both children and adults. Although robotic technology has been adopted for many urologic procedures in adults, only a few studies of RALCCUD have been performed in adults.^13^, ^14^

The median operating time was 313 min. This is comparable with other reports in adults^14^ and children^10^; however, the range is very large (224 to 643 min). We suggest three explanations for this. First, the learning curve as already described by Galanski et al.^10^ showed that operative time decreased from around 500 to 300 min after 10 years of experience. When no concomitant technique is performed, the standardization of the technique makes it shorter. But in our study due to the heterogeneity of the surgery (different type of conduit and concomitant procedures in the first four operations), it is difficult to explore this point. Second, RALCCUD associated with an AC is a real challenge^10^ and requires a much longer operation time. The third reason is when the appendix is not available or not usable. A Casale or Yang‐Monti conduit was used for most patients (66%), requiring an ileal conduit to be used to create the conduit. In our study, the median operative time without concomitant procedures for Mitrofanoff conduit and non‐Mitrofanoff conduit is 244 min (*n* = 3) and 313 min (*n* = 4), respectively.

In most studies, Casale or Yang‐Monti conduits were little used.^10^, ^13^ In our series, the appendix was used in only 33% of patients (4/12). Our results suggest that the creation of a Casale or a Yang‐Monti conduit using robotic assistance is feasible and safe. Furthermore, the short‐term functional results were very good. Indeed, the continence rate was high, and the complications rate was low for all tube types.

In our series, the stomal continence rate without repeat surgery was very high (92%). It was higher than the 60% (6/10 patients) rate reported by Lecoanet et al.^14^ but was similar to rates in other tertiary centres of adult or paediatric urology.^10^, ^13^ We chose to implant the channel posteriorly on the bladder to reproduce de technique initially described by Mitrofanoff.^4^ Although there are no published comparisons of posterior and anterior channels in adults, studies in children suggest that anterior and posterior channels have similar revision rates.^21^ The management of incontinent CCUD with endoscopic injection of submucosal bulking agents is well known to provide good results. Recently, Riachy et al.^22^ found an 86% success rate (either achievement of continence or improvement in continence) in a retrospective study.

The high rate of urethral continence (92%) may have resulted from good preoperative screening and management. In our centre, the intervention was always performed in the neuro‐urology unit with urodynamic assessment. We believe that eligibility screening by a multidisciplinary team including a urologist and rehab physician is key to the success of the intervention.

The rate of complications was low (17%) and was lower than that found in other recent studies in tertiary centres for open, laparoscopic or robot‐assisted surgery.^13^, ^14^ In their comparative study of continent, cutaneous, catheterizable channels, Galanski et al.^10^ reported a complication rate of 43% and 38% for open and robot‐assisted surgery, respectively. Of note, that study was done in children, in whom complications are managed differently from adults.

Only one stomal stenosis occurred during the follow‐up period, and it was managed by endoscopic dilatation. This low rate of stomal stenosis could be attributed to the systematic use of the V‐shaped skin flap to enlarge the circumference of the stoma as previously described.^18^

This study has several limitations. The first is that it was a retrospective, single centre study. However, to our knowledge, it is one the largest studies of RALCCUD in adults to date with 12 individuals included. So far, only two retrospective case‐series have been published, with follow‐ups of less than 24 months.^13^, ^14^ The second limitation is the median follow‐up of only 40.3 months; however, in a retrospective study of 119 stomas, Jacobson et al.^23^ found that stomal stenosis, false passage and first complications occurred within a mean 24.2 months. However, since complications can still occur more than 10 years after the surgery,^7^ long‐term follow‐up is required.

## 5 CONCLUSION

RALCCUD with Mitrofanoff and Yang‐Monti conduits with or without AC seems safe and feasible in adults with NLUTD who are unable to self‐catheterize through the native urethra. It seems to provide a very high short‐term rate of stomal and urethral continence with a low rate of complications. Multicentre, prospective studies are now required to confirm these results and to determine the place of RALCCUD in the management of these issues.

## AUTHOR CONTRIBUTIONS

**Thomas Loubersac**: Protocol/project development; data collection and management; data analysis; manuscript writing/editing. **Etienne Lavallée**: Data analysis; manuscript writing/editing. **Benédicte Reiss**: Data collection and management; manuscript writing/editing. **Marc‐David Leclair**: Manuscript writing/editing. **Pierre Kieny**: Data collection and management; data analysis; manuscript writing/editing. **Marc Le Fort**: Manuscript writing/editing. **Loic Lenormand**: Manuscript writing/editing. **Jerome Rigaud**: Manuscript writing/editing. **Brigitte Perrouin‐Verbe**: Data collection and management; manuscript writing/editing. **Chloé Lefevre**: Manuscript writing/editing. **Marie‐Aimée Perrouin‐Verbe**: Protocol/project development; data analysis; manuscript writing/editing.

## CONFLICT OF INTEREST STATEMENT

The authors declare that they have no conflict of interest.

## Data Availability

The data that support the findings of this study are not openly available due to reasons of sensitivity and are available from the corresponding author upon reasonable request. Data are located in controlled access data storage at Nantes Université, Nantes, France.
